# Factors Associated with Myopia in School Children in China: The Beijing Childhood Eye Study

**DOI:** 10.1371/journal.pone.0052668

**Published:** 2012-12-27

**Authors:** Qi Sheng You, Li Juan Wu, Jia Li Duan, Yan Xia Luo, Li Juan Liu, Xia Li, Qi Gao, Wei Wang, Liang Xu, Jost B. Jonas, Xiu Hua Guo

**Affiliations:** 1 Beijing Institute of Ophthalmology, Beijing Tongren Hospital, Capital Medical University, Beijing, China; 2 School of Public Health and Family Medicine, Capital Medical University, Beijing, China; 3 Beijing Key Laboratory of Epidemiology, Capital Medical University, Beijing, China; 4 Beijing Center for Disease Prevention and Control, Beijing, China; 5 School of Medical Science, Edith Cowan University, Joondalup, Australia; 6 Department of Ophthalmology, Medical Faculty Mannheim, Ruprecht-Karls-University Heidelberg, Heidelberg, Germany; Centre for Eye Research Australia, Australia

## Abstract

**Purpose:**

To assess factors associated with myopia in school children in rural and urban parts of Greater Beijing.

**Methods:**

The Beijing Pedriatic Eye Study was a population-based cross-sectional study, in which one school of each level (primary, junior high, senior high) was randomly selected from nine randomly selected districts out of 18 districts of Greater Beijing. The children underwent non-cylcoplegic refractometry and their parents an interview.

**Results:**

Of 16,771 eligible students, 15,066 (89.8%) children (7,769 (51.6%) girls) participated, with 8,860 (58.8%) participants living in the rural region. Mean age was 13.2±3.4 years (range:7–18 years). In multivariate analysis, prevalence of myopia (defined as ≤−1.00 diopters) was associated with higher age (Odds ratio(OR):1.37; 95% confidence interval(CI):1.35,1.39), female gender (OR:1.35;95%CI:1.25,1.47), key school type (OR:0.77;95%CI: 0.70,0.85), higher family income (OR:1.04;95%CI:1.01,1.07), parental myopia (OR:1.46;95%CI:1.40,1.53), dim reading illumination (OR:0.93;95%CI: 0.88,0.98), longer daily studying duration (OR:1.10;95%CI:1.06,1.15), shorter duration of watching television (or computer) (OR:0.93;95%CI:0.89,0.97), higher self-reported protein intake (OR:0.94;95%CI:0.90,0.99), feeling well about life and status (OR:0.93;95%CI:0.89,0.98), and feeling tired or dizzy (OR:0.94;95%CI:0.91,0.97). Prevalence of high myopia (defined as ≤−6.00 diopters) was associated with higher age (OR:1.43;95%CI:1.38, 1.48), key school type (OR:0.61;95%CI:0.49,0.74), family income (OR:1.07;95%CI:1.02,1.13), parental myopia (OR:1.65;95%CI:1.54,1.76), dim reading illumination (OR:0.86;95%CI:0.77,0.96), less rest during studying (OR:1.18;95%CI:1.10,1.27), feeling well about life and studying (OR:0.88;95%CI: 0.81,0.96) and feeling dizzy or tired (OR:0.93;95%CI:0.87,0.99). Prevalence of high myopia (defined as ≤−8.00 diopters) was significantly associated with higher age (OR:1.39;95%CI:1.31,1.48;), key school type (OR:0.61;95%CI:0.42,0.88) and parental myopia (OR:1.87;95%CI:1.66,2.12).

**Conclusions:**

Myopia in school children in Greater Beijing was associated with higher age, female gender, school type, parental myopia, higher socioeconomic background, dim reading illumination, longer daily studying duration, less rest during study, shorter duration of watching television (or computer), higher self-reported protein intake, feeling well about life and status, and feeling tired and dizzy.

## Introduction

The young generation in East Asian metropolitan regions has witnessed a major increase in the frequency of myopia, with a prevalence of myopia of as high as 80% in the 18-year-old teenagers [1]–[9]. Since myopia, in particular high myopia, can be associated with major ophthalmic diseases such as myopic retinopathy and exudative myopic macular degeneration, myopic glaucomatous optic neuropathy and rhegmatogeneous retinal detachment, studies have addressed potential factors which may be associated with, or which may be even causative for, the development of the myopic shift in the young generation in East Asia [10], [11]. Factors identified to be associated with myopia, in particular in population-based studies from Singapore and Sydney, were older age, female gender, urban region of habitation, type of school, and amount of outdoor activity [12]–[34]. Since these studies were performed mostly on Chinese populations living outside of mainland China, and since the living conditions for children and their parents have been markedly different between mainland China and metropolitan regions such as Singapore and Sydney, and since we were interested to include additional factors potentially associated with the prevalence of myopia, we performed the present investigation on school children from rural and urban regions of Greater Beijing.

## Methods

### Ethics Statement

The ethics committee of the Capital Medical University, the Beijing Municipal Commission of Education and the Beijing Center for Disease Control and Prevention approved the study, and the parents of the children gave written consent.

The Beijing Pediatric Eye Study is a population-based study performed in the region of Greater Beijing and used a stratified cluster sampling technique. The Beijing schools were differentiated into institutions of primary level, junior high level and senior high level. One school from each level was randomly selected from nine randomly selected districts (Xuanwu, Dongcheng, Haidian, Fangshan, Mentougou, Huairou, Changping, Chaoyang, and Tongzhou) out of 18 districts from the rural region and the urban region of Greater Beijing. Greater Beijing is officially divided into a rural region with agriculture still being the predominant source of income, and the urban region. The population from the rural region differs from the population from the urban region by the level of education, profession and income. All students of the selected schools with an age between 7 years and 18 years were invited to participate in the study. Informed consent from at least one parent and each child were obtained before examination.

All selected students and their parents completed a detailed questionnaire with questions on the family status such as number of siblings, uncles, aunts and cousins; questions on family history of ocular diseases; questions on the profession and level of education of the parents, and whether the parents were myopic or not; questions on near work activities such as the usual distance between the eye and the book when reading or writing, the illumination conditions when studying, the distance between the eye and a television set or computer when watching television or using a computer, and the amount of time spent for learning (reading or writing), watching television or working with, or playing on, the computer; questions on type and amount of outdoor exercise, sleeping and diet habits; questions on ocular massage including who taught and controlled the ocular massage and which parts of the ocular regions underwent the self-massage; and questions on the psychological status and diet such as a protein-rich nutrition (Table 1). The interview was carried out by trained school physicians and the quality of the interview was controlled by disease control officers in each district center. The question on parental myopia was phrased as: “Are your parents myopic, e.g., do they wear glasses and can they read without glasses?” After the interview, the children underwent an auto refractometry carried out by a senior experienced optometrist. We used an auto refractor (Topcon RM-A7000; Topcon Co., Tokyo, Japan) and did not apply cycloplegia. The spherical equivalent of the refractive error was calculated as the spherical value of refractive error plus one half of the cylindrical value. Myopia was defined as refractive error (spherical equivalent) of ≤−1.00 diopters, ≤−6.00 diopters and ≤−8.00 diopters, respectively, in the worse eye. The worse eye was defined as the eye with the greater absolute value of refractive error (spherical equivalent).

**Table 1:** Associations between the prevalence of myopia (defined as refractive error ≤−1.00 diopters in the worse eye) and ocular and systemic parameters in the Beijing Pediatric Eye Study.

Parameter	*P*-Value	Odds Ratio	95% Conf. Interval
General Parameters			
Age (Years)***	<0.001	1.33	1.32, 1.35
Gender (Boys/Girls)*	<0.001	1.39	1.31, 1.49
Region of Habitation (Urban/Rural)*	0.07	0.94	0.88, 1.01
Type of School (Key School Yes/No)*	<0.001	0.49	0.45, 0.52
Body Height (cm)***	<0.001	1.01	1.01, 1.01
Body Weight (kg)***	<0.001	1.03	1.02, 1.03
Body Mass Index (kg(m^2^)***	<0.001	1.06	1.06, 1.07
Family Income Per Person (<300 RMB/300-499RMB/500-799RMB/800-1499 RMB/1500-2999RMB/3000-4999 RMB/5000+ RMB)**	<0.001	1.10	1.08, 1.12
Paternal Education (Primary school or less/junior middle school/senior middle school/college/bachelor or postgraduate)**	<0.001	1.13	1.11, 1.16
Maternal Education (Primary school or less/junior middle school/senior middle school/college/bachelor or postgraduate)**	<0.001	1.13	1.11, 1.16
Paternal Profession (Worker/Farmer/Businessman/Health Care, Writer or Drawer, Higher Education/Working in Government)**	<0.001	1.11	1.08, 1.14
Maternal Profession (Worker/Farmer/Businessman/Health Care, Writer or Drawer, Higher Education/Working in Government)**	<0.001	1.11	1.08, 1.14
Parental Myopia (Both Not Myopic/Father Myopic or Mother Myopic/Both Myopic)**	<0.001	1.45	1.37, 1.52
Wearing Glasses (Yes/No)*	<0.001	0.12	0.10, 0.14
Age of Myopia Diagnosis (Years)***	<0.001	1.24	1.21, 1.27
Age at Start of Wearing Glasses (Years)***	<0.001	1.28	1.24, 1.33
How Often Do You Wear Glasses (Always/Often/50-50/Seldom/Never)**	<0.001	0.60	0.56, 0.64
Reading and Studying Conditions			
Illumination during Studying (Table Lamp/Fluorescent Lamp/60-100 Watt Lamp/100+ Watt Lamp)**	<0.001	0.89	0.86, 0.93
Reading Distance (<33 cm/33 cm/>33 cm)**	<0.001	0.64	0.61, 0.68
Daily Study Duration (<8 h/8–10 h/10–12 h/12–13 h/13+ h)**	<0.001	1.50	1.45, 1.55
Active Rest during Studying (Always/Often/Common/Occasionally/Never)**	<0.001	1.12	1.09, 1.15
Reading when Bus Riding (Always/Often/Common/Occasionally/Never)**	<0.001	0.75	0.72, 0.79
Does Your Teacher Correct Your Posture (Always/Often/Common/Occasionally/Never)**	<0.001	1.30	1.26, 1.35
Distance to Computer (<66 cm/66 cm/>66 cm)**	<0.001	0.83	0.79, 0.88
Duration of Television Watching (<1 h/1–2 h/2–3 h/3–4 h/4+h)**	<0.001	0.93	0.90, 0.96
Distance to Television Screen (<2 m/2.0–2.5 m/2.5–3.0 m/3+ m)**	<0.001	0.95	0.92, 0.97
Seat in School (Front Line/Middle Region/Back Row)**	0.03	0.96	0.92, 0.99
Eye Massage			
Frequency (Never/Once/Twice/Three Times/4+ Times)**	0.42	1.02	0.98, 1.06
Quality: Very Skillfully/Skillfully/Less Concentrated/Not Very Interested/Never**	0.27	0.98	0.94, 1.02
Physical Exercise/Diet			
Daily Physical Exercises (No Exercise/<0.5 h/0.5–1 h/1–2 h (2+ h)**	<0.001	0.93	0.90, 0.96
Sleep Duration (<6 h/6–7 h/7–8 h/8–10 h/10+ h)**	<0.001	0.52	0.50, 0.54
Do You Like Sweet Food (Very Much/Quite Like/Common/Occasionally/Never)**	<0.001	1.12	1.09, 1.16
Do You Eat Sweet Food (Very Often/Often/Common/Occasionally/Never)**	0.47	1.01	0.98, 1.05
How Much Vegetables Do You Eat Daily (A Lot/Relatively Much/Common/A Little/Very Little)**	<0.001	0.86	0.83, 0.89
How Much Fruit Do You Eat Daily (A Lot/Relatively Much/Common/A Little/Very Little)**	0.006	0.96	0.92, 0.99
How Much Protein (Including Milk, Egg, Bean, Meat) Do You Eat Daily (A Lot/Relatively Much/Common/A Little/Very Little)**	<0.001	0.90	0.87, 0.94
Psychiatric Status			
How Do You Feel About Your Life and Status (Very Good/Good/Medium/Poor/Very poor)**	<0.001	1.11	1.07, 1.15
Do You Feel Pressure in Life or at School (Very Much/Much/Medium/Little/Very Little)**	<0.001	0.75	0.73, 0.77
Do You Feel Dizzy or Tired (Very Often/Often/Commonly/Occasionally/Rarely)**	<0.001	0.70	0.68, 0.72
What Is Your Mood (Very happy/Happy/Medium/Occasionally Unhappy/Always Unhappy)**	<0.001	1.10	1.07, 1.13
Do You Often Rub Your Eyes (Very Often/Often/Commonly/Occasionally/Rarely)**	<0.001	0.84	0.81, 0.86

* Nominal coding.

** Ordinal coding.

*** Interval coding.

Since involuntary accommodation can influence the refractometric results, a group of students underwent refractometry before and after cylcoplegia. Cycloplegia was achieved by 1% cyclopentolate eye drops, given twice within 10 minutes. If the pupil was not dilated enough (6 mm), a third drop was applied 20 minutes later. Refractometry was performed about 60 minutes after the first instillation of the drops. We measured the difference in refractive error before and after cycloplegia, and calculated the prevalence of myopia using the same definitions as for the main study group.

Using the data of the cycloplegic validation group, we calculated the difference between cylcoplegic refractive error and non-cycloplegic error in the validation group and corrected the refractive error data of the main study group by the values found in the validation group after stratification by age.

Statistical analysis was performed using SPSS for Windows, version 20.0 (IBM-SPSS, Chicago, Illinois, USA). The prevalence was calculated as the number of participants with the particular type of refractive error in relation to the total number of examined children. Prevalence data were given as mean ± standard error. In a first step of the statistical analysis, we examined the associations between the prevalence of myopia and other parameters in a univariate manner, using the *chi-square* test for categorical variables and logistic regression analysis for continuous variables. Categorical variables such as family income, paternal or maternal education level, paternal or maternal profession, parental myopia, and answers to questions such as “How Often Do You Wear Glasses” were analyzed as ordinal variables, since their values were arranged in an ascending or descending order, such as “primary school or less”, “junior middle school”, “senior middle school”, “college”, “bachelor or postgraduate”. In a second step of the analysis, we performed a multivariate binary regression analysis, with the presence or absence of myopia as dependent variable, and the parameters which were significantly associated with the prevalence of myopia in univariate analysis, as independent variables. We first adjusted this regression analysis for the systemic parameters of age, gender, region of habitation, and type of school attended. We then added the other parameters to the regression model. Odds ratios (OR) were calculated and their 95% confidence intervals (CI) were described. All *P*-values were 2-sided and considered statistically significant when less than 0.05.

## Results

Of the 16,771 students eligible and invited to participate the study, 15,066 students participated in the study (response rate 89.8%). They underwent auto-refractometry for both eyes and they and their parents completed the questionnaires. The mean age of the 15,066 participants (7,769 (51.6%) girls) was 13.2±3.4 years (range: 7 to 18 years) (Table 1). Among the study participants, 8,860 (58.8%) were living in the rural region of Greater Beijing, and 6,206 (41.2%) students were living in the urban region; 5,621 (37.3%) children attended the primary school, 4,369 (29.0%) students went to the junior high school, and 5,076 (33.7%) students attended the senior high school. Prevalence of myopia defined as refractive error ≤−1.00 diopters in the worse eye was 57.0±0.4% (95%CI: 56.2, 57.7). In the rural region as compared to the urban region, age was significantly older (13.9±3.4 years versus 12.3±3.3 years; *P*<0.001), refractive error was significantly less myopic (−1.71±2.17 diopters versus −1.87±2.42 diopters; *P* = 0.005), and the level of paternal education (*P*<0.001) and maternal education (*P*<0.001) and the reported family income were significantly higher.

The mean uncorrected refractive error was −1.73±2.34 diopters for the worse eye. After correction by the data obtained in the validation group, the mean corrected refractive error was −1.46±2.29 diopters for the worse eye.

In univariate analysis, prevalence of myopia (defined as refractive error ≤−1.00 diopter in the worse eye) was significantly associated with higher age (*P*<0.001), female gender (*P*<0.001), type of schools (key schools versus non-key schools) (*P*<0.001), higher family income (*P*<0.001), higher paternal and maternal education (*P*<0.001), higher paternal profession (*P*<0.001), parental myopia (*P*<0.001), younger age when myopia was discovered (*P*<0.001), higher prevalence of wearing glasses (*P*<0.001), dimmer illumination conditions when reading (*P*<0.001), longer duration of daily studying (*P*<0.001), higher frequency of active rests during studying (*P*<0.001), higher frequency of reading on bus rides (*P*<0.001), shorter duration of watching television (*P*<0.001), shorter daily physical exercise (*P*<0.001), shorter sleep duration (*P*<0.001), higher intake of protein rich food (*P*<0.001), lower level of emotionally feeling well (*P*<0.001), higher frequency of feeling under pressure (*P*<0.001) or dizzy and tired (*P*<0.001), and higher prevalence of feeling unhappy (*P*<0.001) (Table 1). Prevalence of myopia (≤−1.00 diopters in the worse eye) was not significantly associated with urban region of habitation (*P* = 0.07), frequency (*P* = 0.42) and intensity (*P* = 0.27), of performing an eye massage (*P* = 0.42) (Table 1).

In the first step of a multivariate logistic regression analysis, we used the presence of myopia (≤−1.00 diopters in the worse eye) as dependent parameter and added age, gender, type of school, level of education of father and mother, paternal profession and parental myopia as independent variables. We found that presence of myopia remained to be significantly associated with higher age (OR: 1.39; 95%CI: 1.37, 1.41; *P*<0.001), female gender (OR: 1.39; 95%CI: 1.27, 1.52; *P*<0.001), type of school (key school versus non-key school) (OR: 0.72; 95%CI: 0.64, 0.81; *P*<0.001), higher family income (OR: 1.06; 95%CI: 1.02, 1.09; *P*<0.001), and parental myopia (OR: 1.47; 95%CI: 1.40, 1.54; *P*<0.001). It was no longer significantly associated with the educational level of the father (*P* = 0.07), maternal level of education (*P* = 0.55), and paternal profession (*P* = 0.39). Since region of habitation was associated with the prevalence of myopia in previous studies, we added the region of habitation as parameter to the list of independent variables in the multivariate analysis and found, as in the univariate analysis, that the prevalence of myopia was not significantly associated with the region of habitation (*P* = 0.97) after adjusting for age, gender, school type, family income, and parental myopia.

In a second step of the multivariate analysis, we adjusted for age, gender, type of school, family income, and parental myopia and added step by step in each of the steps one of the remaining parameters which were significantly associated with myopia in the univariate analysis. It showed that prevalence of myopia was associated with dim reading illumination (*P*<0.001), longer duration of daily studying (*P*<0.001), planned rests during studying (*P*<0.001), shorter duration of watching television (*P*<0.001), shorter sleep duration (*P* = 0.02), higher intake of fruits (*P* = 0.02) and protein (*P* = 0.002), liking of sweet food *P* = 0.047), feeling emotionally well (*P* = 0.001) and feeling tired (*P*<0.001) (Table 2).

**Table 2:** Results of the multivariate analysis of the prevalence of myopia (defined as refractive error ≤−1.00 diopters in the worse eye) and ocular and systemic parameters in the Beijing Pediatric Eye Study, after adjusting for age, gender, type of school, family income, and parental myopia, and adding step by step the following parameters.

Parameter	*P*-Value	Odds Ratio	95% Conf. Interval of Odds Ratio
Region of Habitation	0.97		
Body Mass Index	0.47		
Illumination during Studying (Table Lamp/Fluorescent Lamp/60-100 Watt Lamp/100+ Watt Lamp)	<0.001	0.93	0.89, 0.96
Daily Study Duration (<8 h/8–10 h/10–12 h/12–13 h/13+ h)	<0.001	1.15	1.11, 1.20
Active Rest during Studying (Always/Often/Common/Occasionally/Never)	<0.001	1.17	1.13, 1.21
Reading when Bus Riding (Always/Often/Common/Occasionally/Never)	0.62		
Does Your Teacher Correct Your Posture (Always/Often/Common/Occasionally/Never)	0.055		
Duration of Television Watching (<1 h/1–2 h/2–3 h/3–4 h/4+h)	<0.001	0.90	0.87, 0.94
Physical Exercise/Diet			
Daily Physical Exercises (No Exercise/<0.5 h/0.5–1 h/1–2 h (2+ h)	0.16		
Sleep Duration (<6 h/6–7 h/7–8 h/8–10 h/10+ h)	0.02	0.94	0.90, 0.99
Do You Like Sweet Food (Very Much/Quite Like/Common/Occasionally/Never)	0.047	1.04	1.00, 1.08
How Much Vegetables Do You Eat Daily (A Lot/Relatively Much/Common/A Little/Very Little	0.19		
How Much Fruit Do You Eat Daily (A Lot/Relatively Much/Common/A Little/Very Littl	0.02	0.95	0.92, 0.99
How Much Protein (Including Milk, Egg, Bean Meat) Do You Eat Daily (A Lot/Relatively Much/Common/A Little/Very Little	0.002	0.93	0.89, 0.97
Psychiatric Status			
How Do You Feel About Your Life and Status (Very Good/Good/Medium/Poor/Very poor	<0.001	0.93	0.89, 0.97
Do You Feel Pressure in Life or at School (Very Much/Much/Medium/Little/Very Little)	0.13		
Do You Feel Dizzy or Tired (Very Often/Often/Commonly/Occasionally/Rarely	<0.001	0.93	0.90, 0.97
What Is Your Mood (Very happy/Happy/Medium/Occasionally Unhappy/Always Unhappy	0.85		
Do You Often Rub Your Eyes (Very Often/Often/Commonly/Occasionally/Rarely)	0.13		

In a third step of the multivariate analysis, we added all parameters, which were significantly associated with myopia in the second of the analysis, to the list of independent parameters. It revealed that the prevalence of myopia was associated with older age (*P*<0.001), female gender (*P*<0.001), higher school type (*P*<0.001), higher family income (*P* = 0.002), parental myopia (*P*<0.001), dim reading illumination (*P* = 0.005), longer daily studying duration (*P*<0.001), higher frequency of active rests during studying (*P*<0.001), shorter duration of watching television (or computer) (*P*<0.001), higher self-reported protein intake (*P* = 0.02), feeling well about life and study (*P* = 0.002), and feeling tired or dizzy (*P* = 0.001) (Table 3). Prevalence of myopia (≤−1.00 diopters in the worse eye) was no longer significantly associated with level of sleep duration (*P* = 0.32), the intake of fruits (*P* = 0.28), and the liking of sweet food (*P* = 0.12). In the same model, myopia prevalence was neither associated with ocular massage (*P* = 0.97).

**Table 3:** Results of the multivariate analysis of the prevalence of myopia (defined as refractive error ≤−1.00 diopters in the worse eye) and ocular and systemic parameters in the Beijing Pediatric Eye Study.

Parameter	*P*-Value	Odds Ratio	95% Conf. Interval of Odds Ratio
Age (Years)	<0.001	1.36	1.34, 1.38
Gender	<0.001	1.35	1.25, 1.46
School Type (Key School versus no Key School)	<0.001	0.76	0.70, 0.84
Family Income Per Person (<300 RMB/300-499RMB/500-799RMB/800-1499 RMB/1500-2999RMB/3000-4999 RMB/5000+ RMB)	0.002	1.05	1.02, 1.07
Parental Myopia (Both Not Myopic/Father Myopic or Mother Myopic/Both Myopic)	<0.001	1.48	1.42, 1.54
Illumination during Studying (Table Lamp/Fluorescent Lamp/60-100 Watt Lamp/100+ Watt Lamp)	0.005	0.94	0.91, 0.98
Daily Study Duration (<8 h/8–10 h/10–12 h/12–13 h/13+ h)	<0.001	1.11	1.07, 1.15
Active Rest during Studying (Always/Often/Common/Occasionally/Never)	<0.001	1.15	1.11, 1.20
Duration of Television Watching (<1 h/1–2 h/2–3 h/3–4 h/4+h)	<0.001	0.93	0.90, 0.96
Sleep Duration (<6 h/6–7 h/7–8 h/8–10 h/10+ h)	0.32		
How Much Fruit Do You Eat Daily (A Lot/Relatively Much/Common/A Little/Very Little	0.28		
How Much Protein (Including Milk, Egg, Bean Meat) Do You Eat Daily (A Lot/Relatively	0.02	0.94	0.90, 0.99
How Do You Feel About Your Life and Study (Very Good/Good/Medium/Poor/Very poor	0.002	0.93	0.89, 0.97
Do You Feel Dizzy or Tired (Very Often/Often/Commonly/Occasionally/Rarely	0.001	0.94	0.91, 0.97
Do You Like Sweet Food (Very Much/Quite Like/Common/Occasionally/Never)	0.12		

If refractive error (instead of the prevalence of myopia) was taken as independent parameter in a multivariate linear regression analysis, refractive error was significantly associated with higher age (*P*<0.001), female gender (*P*<0.001), higher school type (*P*<0.001), higher family income (*P* = 0.001), parental myopia (*P*<0.001), lower reading illumination (*P*<0.001), longer daily study duration (*P*<0.001), longer duration of watching television (*P* = 0.009), active rest during studying (*P*<0.001), shorter duration of sleep (*P* = 0.002), more protein intake (*P*<0.001), feeling well about life and studying (*P* = 0.02), feeling dizzy and tired (*P*<0.001), and less physical activity (*P* = 0.02) (Table 4). Since region of habitation was associated with the myopic refractive error in previous studies, we added the region of habitation as parameter to the list of independent variables in the multivariate analysis and found, as in the univariate analysis, that the refractive error was not significantly associated with the region of habitation (*P* = 0.07) after adjusting for age, gender, school type, family income, paternal level of education, and parental myopia.

**Table 4:** Linear multivariate regression analysis of the associations between refractive error in the worse eye and other parameters in the Beijing Pediatric Eye Study.

Parameter	*P*-Value	Regr. Coeff. B	Stand. Coeff. Beta	95% Conf. Interval	Variance Inflation Factor
Age (Years)	<0.001	−0.29	−0.42	−0.30, −0.27	1.82
Gender	<0.001	−0.22	−0.05	−0.29, −0.15	1.08
School Type (Key School/No Key School)	<0.001	0.28	0.06	0.19, 0.36	1.43
Family Income Per Person (<300 RMB/300-499RMB/500-799RMB/800-1499RMB/1500-2999RMB/3000-4999 RMB/5000+ RMB)	0.001	−0.04	−0.03	−0.07, −0.01	1.39
Parental Myopia (Both Not Myopic/Father Myopic or Mother Myopic/Both Myopic)	<0.001	−0.69	−0.20	−0.74, −0.63	1.19
Illumination during Studying (Table Lamp/Fluorescent Lamp/60-100 Watt Lamp/100+ Watt Lamp)	<0.001	0 09	0.03	0.05, 0.14	1.03
Daily Study Duration (<8 h/8–10 h/10–12 h/12–13 h/13+ h)	<0.001	−0.08	−0.04	−0.11, −0.04	1.21
Active Rest during Studying (Always/Often/Common/Occasionally/Never)	<0.001	0.12	−0.06	−0.15, −0.09	1.04
Duration of Television Watching (<1 h/1–2 h/2–3 h/3–4 h/4+h)	0.009	0.05	0.02	0,01, 0.08	1.10
Sleep Duration (<6 h/6–7 h/7–8 h/8–10 h/10+h)	0.002	0.08	0.02	0.03, 0.12	1.68
How Much Fruit Do You Eat Daily (A Lot/Relatively Much/Common/A Little/Very Little	0.33				1.15
How Much Protein (Including Milk, Egg, Bean Meat) Do You Eat Daily (A Lot/Relatively Much/Common/A Little/Very Little	<0.001	0.09	0.03	0.05, 0.13	1.17
How Do You Feel About Your Life and Studying (Very Good/Good/Medium/Poor/Very poor	0.02	0.05	0.02	0.01, 0.09	1.19
Do You Feel Dizzy or Tired (Very Often/Often/Commonly/Occasionally/Rarely	<0.001	0.06	0.03	0.03, 0.09	1.27
Daily Physical Exercises (No Exercise/<0.5 h/0.5–1 h/1–2 h (2+ h)	0.02	0.05	0.02	0.01, 0.09	1.10

To address a potentially confounding factor of age on the analysis, we examined which of the variables were significantly associated with myopia risk were additionally associated with age. It revealed that in univariate analysis older age was significantly associated with female gender (*P* = 0.005), lower type of school (*P*<0.001), lower family income (*P*<0.001), less parental myopia (*P*<0.001), longer studying (*P*<0.001), longer watching television (*P*<0.001), less psychological pressure in life and studying (*P*<0.001), less feeling dizzy and tired (*P*<0.001), and more protein intake (*P* = 0.02). In the test of collinearity in the statistical analysis of the associations with myopic refractive error, the variance inflation factor was lower than 2.0 for all parameters included into the study, suggesting that an inter-dependency of the variables did not markedly affect the results of the analysis.

If instead of the uncorrected refractive error data the corrected values were taken for the multivariate linear regression analysis, (myopic) refractive error remained to be significantly associated with higher age (*P*<0.001; regression coefficient B: −0.28 (95%CI: −0.30, −0.27); standardized coefficient beta: −0.42), female gender (*P*<0.001; B: −0.22 (95%CI: −0.29, −0.15); beta: −0.15), higher school type (*P*<0.001; B: 0.27 (95%CI: 0.19, 0.35); beta: 0.06), higher family income (*P* = 0.001; B: −0.05 (95%CI: −0.07, −0.02); beta: −0.03), parental myopia (*P*<0.001; B: −0.67 (95%CI: −0.72, −0.62); beta: −0.20), lower reading illumination (*P*<0.001; B: 0.07 (95%CI: 0.04, 0.10); beta: 0.03), longer daily study duration (*P*<0.001; B: −0.08 (95%CI: −0.11, −0.04); beta: −0.04), shorter duration of watching television (*P* = 0.003; B: 0.05 (95%CI: 0.02, 0.09); beta: 0.02), less active rest during studying (*P*<0.001; B: −0.12 (95%CI: −0.15, −0.09); beta: −0.06), shorter duration of sleep (*P* = 0.001; B: 0.08 (95%CI: 0.03, 0.13); beta: 0.03), more protein intake (*P*<0.001; B: 0.09 (95%CI: 0.05, 0.13); beta: 0.03), feeling well about life and studying (*P* = 0.002; B: 0.06 (95%CI: 0.02, 0.10); beta: 0.02), feeling dizzy and tired (*P*<0.001; B: 0.06 (95%CI: 0.03, 0.09); beta: 0.03), and less physical activity (*P* = 0.03; B: 0.04 (95%CI: 0.01, 0.08); beta: 0.02).

### High Myopia

Prevalence of myopia defined as refractive error ≤−6.00 diopters in the worse eye was 5.2±0.2% (95%CI: 4.9, 5.6). In multivariate analysis, prevalence of high myopia was significantly associated with higher age (*P*<0.001), school type (*P*<0.001), family income (*P* = 0.01), parental myopia (*P*<0.001), dim reading illumination (*P* = 0.009), active rest during studying (*P*<0.001), feeling well about life and studying (*P* = 0.005) and feeling dizzy or tired (*P* = 0.02) (Table 5).

**Table 5:** Results of the multivariate analysis of the prevalence of myopia (defined as refractive error ≤−6.00 diopters in the worse eye) and ocular and systemic parameters in the Beijing Pediatric Eye Study.

Parameter	*P*-Value	Odds Ratio	95% Conf. Interval of Odds Ratio
Age (Years)	<0.001	1.45	1.39, 1.50
School Type (Key School versus no Key School)	<0.001	0.61	0.49, 0.74
Family Income Per Person (<300 RMB/300-499RMB/500-799RMB/800-1499 RMB/1500-2999RMB/3000-4999 RMB/5000+ RMB)	0.01	1.07	1.02, 1.13
Parental Myopia (Both Not Myopic/Father Myopic or Mother Myopic/Both Myopic)	<0.001	1.65	1.54, 1.76
Illumination during Studying (Table Lamp/Fluorescent Lamp/60-100 Watt Lamp/100+ Watt Lamp)	0.009	0.86	0.77, 0.96
Active Rest during Studying (Always/Often/Common/Occasionally/Never)	<0.001	1.18	1.10, 1.27
How Do You Feel About Your Life and Status (Very Good/Good/Medium/Poor/Very poor	0.005	0.88	0.81, 0.96
Do You Feel Dizzy or Tired (Very Often/Often/Commonly/Occasionally/Rarely	0.02	0.93	0.87, 0.99

Prevalence of myopia defined as refractive error ≤−8.00 diopters in the worse eye was 1.3±0.1% (95%CI: 1.1, 1.5). In multivariate analysis, prevalence of high myopia (≤−8.00 diopters) was significantly associated with higher age (*P*<0.001), school type (*P* = 0.009) and parental myopia (*P*<0.001) (Table 6).

**Table 6:** Results of the multivariate analysis of the prevalence of myopia (defined as refractive error ≤−8.00 diopters in the worse eye) and ocular and systemic parameters in the Beijing Pediatric Eye Study.

Parameter	*P*-Value	Odds Ratio	95% Conf. Interval of Odds ratio
Age (Years)	<0.001	1.36	1.28, 1.44
School Type (Key School versus no Key School)	0.006	0.59	0.41, 0.86
Parental Myopia (Both Not Myopic/Father Myopic/Mother Myopic/Both Myopic)	<0.001	2.57	2.13, 3.12

### Validation Study

The participants of the validation study were a sub-group of the main study population. All children, participating in the study, and their parents were explained the side effects of cycloplegia. Those children who were willing and who were allowed to undergo cycloplegia took part in the validation study. The validation study group included 1082 children (541 (50%) girls) with a mean age of 10.4±2.3 years (median: 10 years; range: 7–18 years). The validation study group was significantly (*P*<0.001) younger than the main study group, while both groups did not vary significantly in gender. The children of the validation study underwent cycloplegic refractometry with the same auto refractor and by the same optometrist as the other study participants.

**Figure 1:**
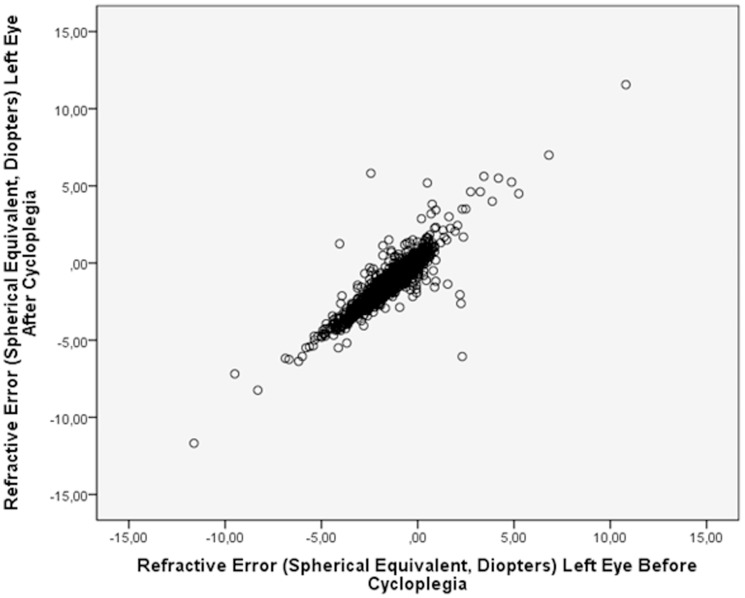
Scattergram showing the distribution of refractive error before and after cycloplegia in 1082 children with a mean age of 10.4±2.3 years.

In the validation study group, mean refractive error was prior to, and after, cycloplegia −1.60±1.46 diopters and −1.26±1.62 diopters, respectively in the right eyes, and it was −1.43±1.50 diopters and −1.12±1.64 diopters, respectively in the left eyes (Fig. 1). The difference in refractive error between the status prior to, and the status after cycloplegia was 0.31±0.47 diopters for the right eyes and it was 0.34±0.46 diopters for the left eyes. The difference between pre-cylcoplegic values and post-cylcoplegic values increased significantly with the pre-cylcoplegic hyperopic refractive error (right eyes: correlation coefficient r = 0.20; *P*<0.001; left eyes: r = 0.28; *P*<0.001). The difference between pre-cylcoplegic values and post-cylcoplegic values was 0.57±0.63 diopters for the group of eyes with a refractive error ranging from hyperopia to myopia of ≤−0.50 diopters, and it was 0.29±0.40 diopters for the group of eyes with a myopic refractive error of more than −0.50 diopters.

## Discussion

In our population-based study on a study population of more than 15,000 children in Greater Beijing, the prevalence of myopia (≤−1.00 diopters in the worse eye) and myopic refractive error were significantly (*P*<0.05) associated with higher age, female gender, key school type, higher family income, parental myopia, dim reading illumination, longer daily studying duration, shorter duration of watching television (or computer), higher self-reported protein intake, feeling well about life and status, and feeling tired, and less physical activity (Tables 3, 4).

Our study confirms previous investigations on young Chinese populations from Singapore and Sydney, that older age, female gender, parental myopia [12], [24]–[26], near work [17], [18], [20], [24], [29], school achievements and other school associated factors [13], [16], [23], and higher intensity in studying (as expressed by the parameters of attended school type and daily duration of studying) were factors which were independently associated with a higher prevalence of myopia and with a higher myopic refractive error in children. Similar findings have been reported by Saw, Low and colleagues from Singapore [5], [14], [16]–[18], Rose and coworkers from Sydney and Singapore [13], Mutti et al. [23], Jones et al. [24],and Ip and colleagues [28], to mention a few. Besides the genetic factor in the form of parental myopia, it suggests that the intensity of studying is a major environmental and behavioral factor for the development of myopia.

Interestingly, dim illumination during reading was an additional factor which independently of parental myopia and intensity of studying was constantly associated with a higher prevalence and amount of myopia (Tables 1, 2,3, 4, 5, 6). It is in agreement with a recent article by Rose and colleagues who suggested that bright light outdoors might prevent myopia by increasing the release of dopamine from the retina, since dopamine has been known to be an inhibitor of axial elongation [35], [36]. It was shown that bright light prevented the development of myopia in animal models and that this prevention was dopamine-dependent [37]. The results of these experimental studies supported the findings obtained in studies by Rose, Guggenheim and others of an association between outdoor activity and myopia since outdoors activity is associated with the exposure to bright light [38], [39]. The hypothesis of an association between dim light and myopia may be strengthened by a recent study by French and colleagues who found that European Caucasian children in Northern Ireland (with less sun light) had a greater prevalence of myopia (as well as of hyperopia and astigmatism) when compared to children living in Sydney (with more intensive sun light) [40]. The finding of an association between dim illumination during reading and myopia in our study may be in contrast to a study by Loman and colleagues [27], who examined 179 law students and found that the strongest association, especially in those subjects with myopia onset before college, was a relation between myopia progression during law school and less daily exposure to darkness. In the study by Loman, however, hours of darkness were mostly those spent during sleeping, since most study participants had the lights switched off during sleeping. Then, however, the time of darkness in Loman's study corresponds to the time of sleeping in our study, with a short time of sleeping associated with a higher prevalence and amount of myopia in our study (Table 1, 2). Another question is whether the association between dim reading illumination and myopia was a causal relationship or a parallel association. It could be that myopic subjects deliberately choose a dimmer reading light because due to their myopic optical system, reading in dimmer light may be easier than in bright light. If further investigations confirm our findings on an association between dim reading illumination and myopia and prove a causal relationship, increased illumination during reading could eventually be a method against the development of myopia in school children.

As in previous studies, the amount of myopia was associated with less physical activity which usually is performed outdoors [4], [19], [20], [21], [29]. It confirms recent studies by Rose and colleagues who found that a lower prevalence of myopia in Chinese children raised in Sydney as compared to Chinese children living in Singapore was associated with increased hours of outdoor activities [13], [29]. Rose and colleagues were the first to separate the effects of being outdoors and being physically active on the association with myopia. Rose and coworkers additionally hypothesized that another factor contributing to the differences in the prevalence of myopia between Chinese children from Sydney versus Singapore was the early educational pressures found in Singapore but not in Sydney. Rose's study was supported by an investigation of Guggenheim and colleagues who measured physical activity objectively and who found that both less time spent outdoors and less physical activity were associated with incident myopia, with time outdoors having the larger effect [38]. Guggenheim and coworkers concluded that time spent outdoors was predictive of incident myopia independently of the physical activity level. Mutti and coworkers reported a protective effect of outdoor activities against myopia, in both a cross-sectional study [23], and a longitudinal study [24]. It also agrees with the recent study by Jones-Jordan and colleagues who found that before the onset of myopia, near work activities of future myopic children did not differ from those children who remained emmetropic [41]. Those children who became myopic had fewer outdoor/sports activity hours than the remaining emmetropic children before, at, and after myopia onset. Jones-Jordan concluded that myopia onset might influence children's near work behavior, but the lack of difference before onset argued against a major causative role for near work; and that less outdoor/sports activity before myopia onset might exert a stronger influence on development than near work. The finding of an association between outdoors activity and myopia was also reported for other ethnic groups, such as from Jordan and from Turkey [32], [33]. One may conclude that besides differences in cultural parameters, the physical outdoor activity could play a role in the development of myopia in children, independent of their ethnicity. Since the parameter of outdoor activity, in contrast to gender or age, can be influenced, our study in agreement with the previous investigations suggests that an increase in outdoor activities may potentially be a protective factor against myopia in school children.

Interestingly, prevalence and amount of myopia was associated with the self-reported intake of proteins such as milk, egg, beans and meat (Tables 1, 2, 3, 4). This finding may be parallel to the results of study by Lim and colleagues [31], who examined 851 Chinese schoolchildren from Singapore and found that axial length was longest in the highest quartile group of total cholesterol intake compared with the lowest (*P* = 0.03) and was longest in the highest quartile group of saturated fat intake compared with the lowest (*P* = 0.04). None of the nutrients, however, was associated with refractive error or a diagnosis of myopia. While the data of Lim's study may suggest that children with a higher cholesterol intake may have larger globes, potentially in associated with a taller body height [42], the finding of our study may make raise the question whether the association between protein intake and myopia may be just another expression of the association between myopia and educational and socioeconomic background of the parents.

Potential limitations of our study should be mentioned. The most important limiting factor in our relatively large scaled study was that cycloplegia was not performed. This might lead to over-estimation of myopia and under-estimation of hyperopia due to accommodation. To partially overcome this weakness in the study design, we performed a validation study on a second group of students of 1082 children. The difference between pre-cylcoplegic values and post-cylcoplegic values increased significantly with the pre-cylcoplegic hyperopic refractive error (*P*<0.001). It was 0.57±0.63 diopters in the range from hyperopia to a myopic refractive error of −0.50 diopters, and it was 0.29±0.40 diopters in the eyes with a myopic refractive error of more than −0.50 diopters. Since we used a definition of myopia of ≤−1.00 diopters in the worse eye, the lack of cylcoplegia may therefore not have markedly influenced the results of our study. One has to consider however, that the population of the validation study was significantly (*P*<0.001) younger than the main study group, so that the results may not directly be transferable to the main study population. Interestingly, however, if the non-cycloplegic refractive error data were age-adjusted corrected on the basis of the validation study, the same parameters were associated with myopia. It may indicate that the major weakness of our study, i.e., the failure to perform cycloplegic refractometry in the main study population, may not have markedly influenced the results of the investigation. In addition, one may consider that the goal of the study was not to assess the prevalence of myopia but to search for factors which are associated with the refractive error. Second, the region of Greater Beijing may not be representative for whole China. Although our study included an urban part and a rural region, the level of education and the socioeconomic parameters were higher in the rural part of our study than in rural regions of other provinces of China. Third, our investigation was a cross-sectional study which does not allow drawing conclusions on a longitudinal course and causal relationship between parameters.

In conclusion, our study showed that myopia in school children in Greater Beijing was associated with higher age, female gender, higher school type, parental myopia, higher socioeconomic background, dim reading illumination, longer daily studying duration, shorter duration of watching television (or computer), higher self-reported protein intake, feeling well about life and status, feeling tired and dizzy, and less physical activity.
